# Assessing the Stability of Herbicide-Tolerant Lentil Accessions (*Lens culinaris* Medik.) under Diverse Environments

**DOI:** 10.3390/plants12040854

**Published:** 2023-02-14

**Authors:** Rind Balech, Fouad Maalouf, Somanagouda B. Patil, Karthika Rajendran, Lynn Abou Khater, Diego Rubiales, Shiv Kumar

**Affiliations:** 1 International Center for Agricultural Research in the Dry Areas (ICARDA), Terbol 1108-2010, Lebanon; 2 ICARDA, Rabat Institutes, Rabat P.O. Box 6299, Morocco; 3 VIT School of Agricultural Innovations and Advanced Learning (VAIAL), Vellore Institute of Technology, Vellore 632014, India; 4 Institute for Sustainable Agriculture, CSIC, 14004 Córdoba, Spain; 5 ICARDA, New Delhi 110012, India

**Keywords:** lentil, post-emergence herbicide, herbicide tolerance, imazethapyr, metribuzin, stability parameters, GGE biplot

## Abstract

Assessing the adaptability and stability of herbicide-tolerant lentil accessions to two broad-spectrum post-emergence herbicides in multi-environment trials has become a must in a breeding program to improve its selection. The adaptability and stability of 42 herbicide-tolerant lentil accessions were investigated using five stability parameters under eight different environments. Significant Genotype–Environment (GE) interaction was found for days to flowering (DFLR), days to maturity (DMAT), and seed yield per plant (SY). The analyzed stability parameters such as Cultivar superiority, Finlay–Wilkinson, Shukla, Static Stability, and Wricke’s Ecovalence ranked the tested accessions differently, confirming the importance of using a combination of stability parameters when evaluating the performance of a group of accessions. GGE biplot of the SY trait accounted for 60.79% of sums of squares of the GE interaction and showed that cool and high rainfall environments are ideal for testing the agronomic performance of tolerant accessions. The GGE biplot of SY showed that IG4605(19), IG195(6), and IG156635(12) were specifically adapted to one mega environment, whereas IG70056(38) was identified as a superior line having a high and stable yield. These lines should be included in lentil crossing programs to develop herbicide-tolerant cultivars adapted to diverse environments.

## 1. Introduction

Lentil (*Lens culinaris* Medik.) is one of the oldest annual crops in the world, as old as the domestication of einkorn, emmer, barley, and peas for cultivation. It originated near southeastern Turkey and Syria around 7500 BC and spread over the near east, Egypt, Central and Southern Europe, the Mediterranean basin, Ethiopia, Afghanistan, India, Pakistan, China, and eventually to Latin America [1]. It is a cool-season legume crop that plays a major role in human nutritional security due to its high protein content (20–36%), carbohydrate (60–67%), lipid (<4%), and ash (2–3%) on a dry basis [2], in animal feeding, and in soil health and is an essential component for crop rotation, particularly with cereals [3]. Currently, Canada (39.2%), India (19.4%), Australia (6.5%), Turkey (6.5%), the United States of America (5.0%), Nepal (4.0%), China (2.6%), and Ethiopia (2.4%) are the leading producing countries of lentils [4].

Besides the importance stated above, there is a need to increase the productivity of lentils in many countries where it is subjected to severe biotic and abiotic stresses. Weeds are one of the most damaging biotic stresses to lentil productivity causing severe yield losses of up to 95% in North Africa and Western and Central Asia [5,6]. Weeds compete with lentils throughout their life cycle due to their shallow roots, poor early vigor, and slow vegetative growth, especially during the cool season [7]. Pre-emergence herbicides are effective in controlling weeds early in crop growth, but weeds germinating after crop emergence at the pre-flowering stage pose a threat to lentil production [8]. Weeds can be controlled using mechanical and manual weeding, soil sterilization, and high seed rate density [9], but these methods are either inefficient or very expensive [10]. Post-emergence herbicides like imazethapyr and metribuzin are effective at controlling weeds in many legume crops, including lentils [6,11], chickpeas [8], and soybeans [12]. These herbicides can control a broad spectrum of annual and parasitic weeds [13], but they are phytotoxic to existing lentil genotypes. Therefore, it is necessary to develop herbicide-tolerant lentil cultivars with stable yields under a variety of conditions [6,7,11,14]. However, the wide adaptability of these accessions must be proven in order to include them in commercial farming at a large scale [15]. Therefore, they must be tested under different environments to assess their yield stability.

The performance of a genotype depends on the genotypic value (G), environmental effect (E), and GE interaction. Yan et al. [16] suggested G and GE effects instead of only GE interaction for yield stability analysis. The ranking of different genotypes defines GE interaction under various environmental conditions [17] by measuring its plasticity. Additionally, it identifies the most suitable test environments, allocates resources within a breeding program, and assists with the selection of germplasm and breeding strategy [18].

The stability of any genotype suggests that E and GE interaction does not change its ranking and performance. In lentils [19], maize [20], and grass peas [21], several stability analyses have been used to determine if the tested genotypes are stable. GGE biplot allows visualizing the which-won-where pattern and displays the interrelationships among all test environments. This method allows ranking the genotypes based on yield and stability performance. Since grain yield is the most affected trait in many crops (references), we focused on evaluating the yield of lentil accessions in our study. The stability of herbicide-tolerant accessions in fava beans has previously been reported [22].

Consequently, the primary objectives of this study were (1) to evaluate the performance of lentil accessions under imazethapyr and metribuzin treatments, (2) to assess the yield stability of these accessions across a variety of environments, and (3) to identify the ideal environment for selection.

## 2. Results

### 2.1. Crop Phenology

The combined variance analysis revealed *p* < 0.001 among the 42 accessions (G) across the test environments (E) and their interaction (GE) for days to flowering (DFLR) and days to maturity (DMAT), indicating that the genotypes behaved differently under diverse environments. For both traits, the combined narrow sense heritability (h^2^) was approximately equal to 0.9 (Table 1).

The analysis of variance in DFLR and DMAT conducted independently for each environment showed significant differences (*p* < 0.001) among accessions in all environments with the exception of DMAT at Marchouch-2015/16-metribuzin at 210 (g a.i. ha^−1^) (E0), Marchouch-2015/16-no herbicide treatment (E1), Terbol-2018/19-metribuzin at 210 (g a.i. ha^−1^) (E6), and Terbol-2018/19-no herbicide treatment (E7) (Table 2). For DFLR, the estimated narrow sense heritability (h^2^) ranged from 0.35 in (E0) to 0.87 in (E5), and for DMAT, it ranged from 0.0 in (E0) and (E1) to 0.7 in (E3) (Table 2).

In environments treated with imazethapyr or metribuzin, we found that DFLR was delayed in all accessions. The earliest flowering was observed in environments (E1, E4, and E7) untreated with herbicides, where the average DFLR was 99.6, 62.8, and 95.8 days. The widest range in DFLR among accessions was observed at Terbol-2018/19-imazethapyr at 75 (g a.i. ha^−1^) (E5), where DFLR fluctuated between 90.6 and 118.7 days after sowing (DAS), and the narrowest range in DFLR was observed at Marchouch-2016/17-imazethapyr at 75 g (g a.i. ha^−1^) (E2) (Table 3).

**Figure 1:**
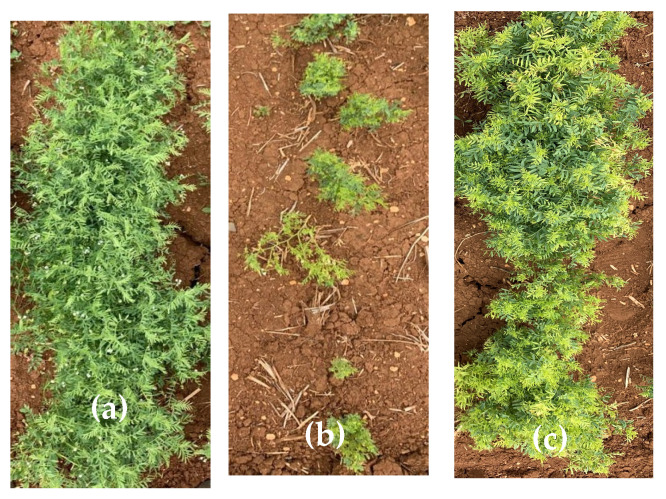
IG115370 (**a**) plot with no herbicide treatment; (**b**) plot treated with imazethapyr; (**c**) plot treated with metribuzin.

Similar to DFLR, DMAT was delayed in environments treated with imazethapyr or metribuzin except for Marchouch-2015/16-metribuzin at 210 (g a.i. ha^−1^) (E0) (Figure 1a–c). The widest range in DMAT was observed at Marchouch-2015/16 (E0) and (E1), followed by Terbol-2018/19-no herbicide treatment (E7), whereas the range of DMAT was limited at Terbol-2018/19-imazethapyr at 75 g a.i. ha^−1^ (E5) (Table 3).

### 2.2. Yield Attributes

The combined analysis for biological yield per plant (BY), seed yield per plant (SY), number of pods per plant (NPP), and number of seeds per plant (NSP) revealed a significant (*p* < 0.001) variation between genotypes (G), environments (E) and the GE interaction of the eight environments, except for GE interaction of BY (*p* = 0.51) and NPP (*p* = 0.15). This demonstrates that the genotypes of SY and NSP responded differently to various environmental conditions (Table 1).

The average narrow sense heritability (h^2^) of BY was 0.21, ranging from zero at Marchouch-2015/16-metribuzin at 210 (g a.i. ha^−1^) (E0) and Terbol-2018/19-no herbicide treatment (E7) to 0.5 at Marchouch-2016/17-imazethapyr at 75 g (g a.i. ha^−1^) (E2) (Table 2). The analysis of the variance in BY for each environment revealed that genotypes responded differently to different environments (*p* < 0.05), with the exception of Marchouch-2015/16-metribuzin at 210 (g a.i. ha^−1^) (E0) (*p* = 0.062), Terbol-2018/19-metribuzin at 210 (g a.i. ha^−1^) (E6) (*p* = 0.053) and Terbol-2018/19-no herbicide treatment (E7) (*p* = 0.081) (Table 2). When no herbicide treatment was applied, the average BY was higher than when imazethapyr or metribuzin were applied. At Marchouch-2016/17-no herbicide treatment (E4), the BY was 5.12 g, followed by 4.79 g at Terbol-2018/19-no herbicide treatment (E7) and 2.41 g at Marchouch-2015/16-no herbicide treatment (E1). This is then followed by the average BY of environments treated with metribuzin (E3 and E6) and imazethapyr (E2 and E5).

The average narrow sense heritability (h^2^) of SY was 0.57, ranging from 0.001 in (E0) and (E1) to 0.72 during Marchouch-2016/17-imazethapyr at 75 (g a.i. ha^−1^) (E2), indicating that each accession responded differently to the various combinations of environments (Table 2). The highest values for narrow sense heritability (h^2^) were observed for accessions E2 (0.72), E3 (0.69), E4 (0.58), E5 (0.53), E6 (0.51) and E7 (0.31), indicating repeatability that the trait is replicable among accessions exposed to various herbicides.

The analysis of the variance in SY for each environment revealed that genotypes responded differently to various test environments (*p* < 0.001), except for Marchouch-2015/16 (E0 and E1) (Table 2). The average SY at Terbol 2018/19, with no herbicide treatment, was the highest (E7), followed by metribuzin 210 (g a.i. ha^−1^) (E6) and then imazethapyr 75 (g a.i. ha^−1^) (E5). A similar observation was made for Marchouch 2016/17 environments (E2, E3, and E4). Comparing the environments treated with imazethapyr (E2 and E5), Marchouch-2016/17-imazethapyr at 75 (g a.i. ha^−1^) had a higher average SY (E2). When comparing the environments treated with metribuzin at Marchouch (E3) and Terbol (E6), the response of the genotypes in both environments was identical (Table 3).

### 2.3. Stability Analysis

Significant GE interaction for the SY resulted in the estimation of five stability parameters along with their rankings, which are presented in Table 4. At each parameter level, the accessions with the lowest values were considered the most stable. According to the Cultivar superiority index, IG195 is the most yielding and stable line. The most stable accession was ILX87075 based on the static stability index, IG69492 based on Wricke’s Ecovalence and Shukla, and IG114670 based on Finlay–Wilkinson.

To statistically compare the five stability parameters, Spearman’s coefficient of rank correlation was calculated; it ranged from −0.6 and 0.87, indicating a wide range of variation in the performance of the accessions across the parameters. There was a highly significant but negative correlation between cultivar superiority, Finlay–Wilkinson (−0.57), and between cultivar superiority and static superiority (−0.60). Conversely, highly significant and positive correlations existed between Shukla and Wricke’s Eco-valence (0.87), which identified eight stable accessions: IG114663, IG115370, IG156514, IG257, IG5244, IG69492, IG75929, and IG76251. Furthermore, a positive correlation existed between Static Stability and Finlay–Wilkinson identifying three stable accessions: IG114663, IG257, and IG75929. Four stable accessions were identified using Static stability and Shukla: IG114663, IG114670, IG257, and IG75929, with Static stability and Wricke’s Ecovalence identifying three stable accessions: IG257, IG75929, and IG114663. IG114663, IG257, and IG75929 were the most stable genotypes as they ranked among the top ten most stable genotypes based on a variety of parameters. Nevertheless, the rankings of the identified stable genotypes vary from one parameter to another despite their positive correlation (Table 5).

### 2.4. GGE-Biplot

**Figure 2:**
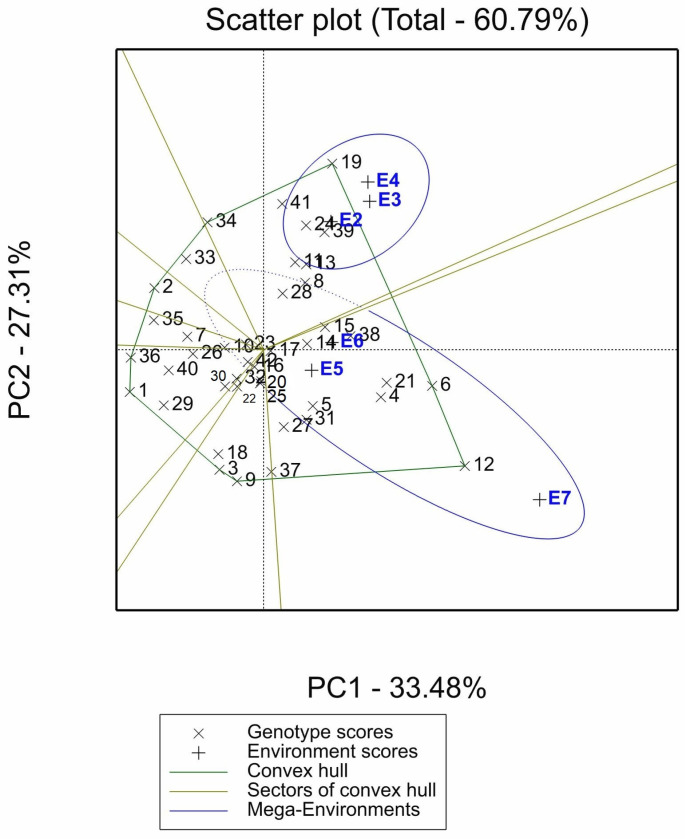
GGE biplot of tested accessions in validation trials for yield data (SY) explained 60.40% of the total variability. E2: Marchouch-2016/17-imazethapyr at 75 g a.i. ha^−1^, E3: Marchouch-2016/17-metribuzin at 210 g a.i. ha^−1^, E4: Marchouch-2016/17-no herbicide treatment, E5: Terbol-2018/19-imazethapyr at 75 g a.i. ha^−1^, E6: Terbol-2018/19-metribuzin at 210 g a.i. ha^−1^ and E7: Terbol-2018/19-no herbicide treatment. Accessions numbered 1 to 42 are listed in Table 5.

A GGE biplot was conducted for seed yield per plant (SY) traits to assess the reproducibility of the tested lentil accessions and determine the which-won-where pattern. The biplot accounted for 60.79% of the variation (Figure 2). Environments E0 and E1 were omitted from the GGE biplot analysis due to low heritability and, consequently, low variability, which may not be due to genetic variation but rather to environmental conditions.

GGE biplot revealed that environments E2, E3, and E4 were highly correlated, as were environments E5, E6, and E7. However, E4 and E7 have the weakest correlation and the greatest angle between their vectors.

As the GGE biplot provides an indication of the discriminating ability of each test environment based on the vector length, the E7 environment was the most discriminating for the tested genotypes, whereas the E5 environment was the least discriminating.

The GGE biplot also displays a polygon view depicting the distribution of genotypes, with some genotypes located on the polygon’s vertex and located within it. The genotypes located on the polygon’s vertex are the farthest ones from the biplot’s origin compared to those located on the polygon’s similar sectors. Therefore, they are considered the most responsive ones. The genotypes located on the vertex were IG1455, IG2445, IG257, IG195, IG857, IG156635, IG4605, ILL8009, and ILX87075 (1, 2, 3, 6, 9, 12, 19, 34, and 36). The genotypes IG1455, IG2445, IG257, IG857, ILL8009, and ILX87075 (1, 2, 3, 9, 34, and 36) were not considered winning genotypes in any of the test environments because no environments were located within the sectors of the previously mentioned vertex genotypes.

The GGE biplot also identifies the mega-environments within each; multiple environments, as well as their winning genotypes, reside within each mega-environment. The GGE biplot of the SY was subdivided into nine sectors and two mega-environments (ME) located in two different sectors. Mega environment 1 (ME1) consisted of Marchouch-2016/17-imazethapyr at 75 (g a.i. ha^−1^) (E2), Marchouch-2016/17-metribuzin at 210 (g a.i. ha^−1^) (E3), and Marchouch-2016/17-no herbicide treatment (E4). Mega-environment 2 (ME2) consisted of Terbol-2018/19-imazethapyr at 75 (g a.i. ha^−1^) (E5), Terbol-2018/19-metribuzin at 210 (g a.i. ha^−1^) (E6), and Terbol-2018/19-no herbicide treatment (E7).

The GGE biplot analysis of the SY revealed that genotype IG4605 (19) was the winning genotype in the ME1, having the highest seed yield per plant, while genotypes IG195 (6) and IG156635 (12) had the highest seed yield per plant in the ME2.

### 2.5. Yield Components Ranking and Stability of Genotypes

**Figure 3:**
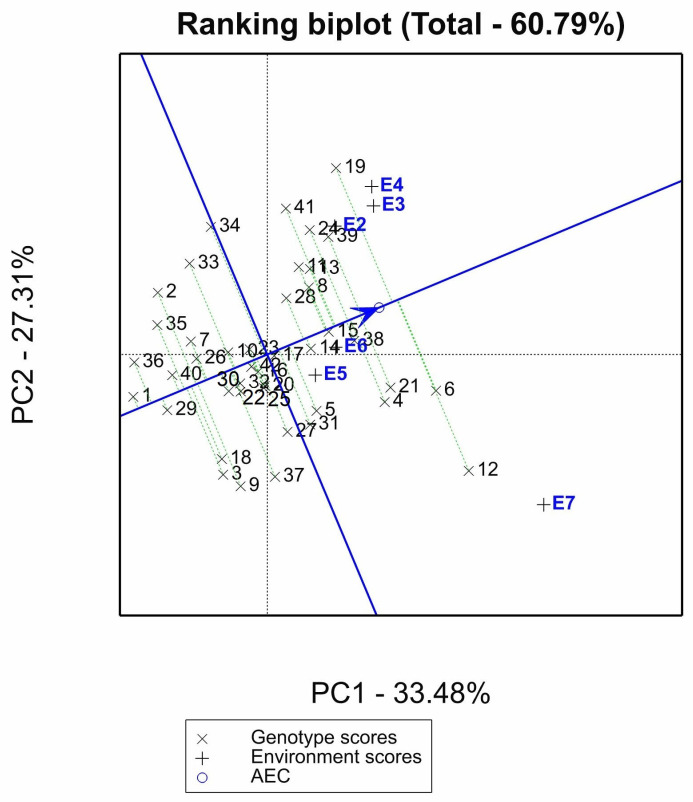
Average environment coordination (AEC) views of the GGE biplot show the mean yield performance and genotype stability. PC: principal component, E2: Marchouch-2016/17-imazethapyr at 75 g a.i. ha^−1^, E3: Marchouch-2016/17-metribuzin at 210 g a.i. ha^−1^, E4: Marchouch-2016/17-no herbicide treatment, E5: Terbol-2018/19-imazethapyr at 75 g a.i. ha^−1^, E6: Terbol-2018/19-metribuzin at 210 g a.i. ha^−1^ and E7: Terbol-2018/19-no herbicide treatment. Accessions numbered 1 to 42 are listed in Table 5.

The mean environment coordination method (MEC) of this study showed that 18 genotypes were located on the right side of the mean environment ordinate, indicating that their seed yield per plant was greater than the average, whereas 24 genotypes’ seed yields were less than the average. The highest-yielding genotypes were IG195, IG156635, and IG4605 (6, 12, and 19), while the lowest-yielding genotypes were IG1455, IG114663, and ILX87075 (1, 29, and 36). Based on the parallel projections shown in Figure 3, IG156771 (15) was the most stable and had the nearest projection to the mean environment axis, whereas IG156635 (12) had the farthest projection from the mean environment axis. Accessions IG590 (8), IG156656 (14), IG156771 (15), IG4400 (17), IG76251 (28), IG70056 (38), and 2009S 96568-1 (39) had yields that were higher than or comparable to the average environment and were deemed to be relatively stable.

## 3. Discussion

Weeds are a major concern for developed and modernized farming systems that employ a small number of workers. Commercial lentil crop expansion requires machine-harvestable varieties with appropriate weed management practices. Furthermore, cultivar development necessitates consistency across environments, as several studies have shown that these could have multivariate responses to different environments [15,23]. Therefore, in order to integrate lentils into the modernized cereals-based system, it is necessary to develop lentil cultivars that are tolerant to post-emergence herbicides and adaptable to a wide range of environments. In multi-environment trials, the performance and stability of breeding lines can be evaluated in order to identify the ideal environments for lentil screening, characterize mega environments, and detect accessions with specific and broad adaptation [24,25].

### 3.1. Phenological Traits

Herbicide treatment with imazethapyr and metribuzin delayed flowering and maturity in lentils, which is consistent with previous research in lentils [7], chickpeas [8], and fava beans [26]. This delay in maturity was explained by Gaur et al. [8] as a slowdown in the crop growth rate occurred after herbicide treatment due to starvation and blockage in acetolactate synthase catalyzed reactions [27]. Furthermore, herbicide-tolerant fava beans [26] and lentils [7] accessions were affected by the herbicide treatment, but subsequent plant growth led to recovery, resulting in further delay of flowering and maturity time.

The flowering and maturity times of lentil accessions were longer at Terbol than at Marchouch. This finding is explained by the fact that the climate at Terbol is cooler and has more precipitation than the climate at Marchouch, as reported in fava beans by Abou-Khater et al. [22]. Furthermore, heat and drought stress have been shown to shorten crop cycle duration in lentils [28], chickpeas [29], and fava beans [30]. The delayed flowering and maturity observed in both treatments at Marchouch 2015/16 was expected due to an exceptional season with a lower-than-usual maximum temperature.

### 3.2. Yield Attributes

Seed yield was lower in environments treated with imazethapyr or metribuzin than in environments not treated with herbicides. Similar findings have been previously made in lentils [6,7], fava beans [26], and chickpeas [31]. Furthermore, biological yield per plant (BY) at Marchouch in 2015/16 was lower than at Terbol in 2018/19, which was followed by Marchouch in 2016/17 due to low precipitation in January at Marchouch in 2015/16 and well-distributed precipitation from December to February at Marchouch in 2016/17 during the vegetation growth phase. For SY, the highest value was obtained at Terbol in 2018/19 with no herbicide treatment (E7), which was expected given that this environment experienced high precipitation and low temperatures and no herbicide treatment during the crop season.

The heritability estimate from the multi-environment trial analysis is more accurate than the estimates from a single environment. Heritability estimates for phenological traits (DFLR and DMAT) were higher than growth and yield attributes (PH, BY, and SY). Lower heritability estimates for BY and SY indicated that these traits were highly influenced by environmental factors and controlled a large number of genes with a small effect when compared to phenological traits. These findings are consistent with previous research on fava beans [22], chickpeas [32], and lentils [33].

### 3.3. Stability Parameters

Stability parameters are used to assess genotype performance in terms of yield and stability in a variety of environments [33]. In the current study, five stability parameters were used to rank the genotypes in terms of stability. Previous research on lentils and other crops compared stability parameters to advise the breeders on the best method to use for selection. Our findings revealed inconsistencies in genotype ranking, as previously reported in fava beans [22], lentils [33,34], chickpeas [35], and sorghum [36]. Nevertheless, the analysis of Spearman’s coefficient revealed that there were some correlations between these stability parameters. Dehghani et al. [23] made similar observations about the similarity between Wricke’s Ecovalence and the Shukla parameters, but they disapproved the similarity between Finlay and Wilkinson and Static Stability. Furthermore, several studies have confirmed the ability of the cultivar superiority index to select genotypes with high and stable yields [37,38]. The most stable genotypes were identified using the static stability, Wricke’s Ecovalence, Shukla, and Finlay–Wilkinson parameters across all test environments [39,40]. However, our study found that the cultivar superiority parameter was not related to any of the other parameters studied and was also negatively correlated with the Static Stability and Finlay–Wilkinson parameters. Abou-Khater et al. [22,41] obtained similar observations. In our study, three accessions, IG257, IG75929, and IG114663, were identified as the most stable genotypes using the static stability, Wricke’s Ecovalence, Shukla, and Finlay–Wilkinson parameters, as well as being moderately to highly tolerant to imazethapyr and metribuzin. The cultivar superiority parameter, however, ranked these genotypes among the least stable. As a result, selecting stable and high-yielding genotypes would necessitate the use of more than one parameter [42].

### 3.4. GGE Biplot, Ranking, and Comparison with Stability Parameters

Breeding lines with a narrow genetic base are typically less stable than those with a broad genetic base [43]. Stable genotypes are well adapted to a wide range of environments, whereas unstable genotypes have limited adaptability. A genotype is considered stable if it contributes little to GE interaction [39]. Environmental conditions have been shown to influence herbicide response in fava beans [22], soybeans [44], and corn [45].

In this study, the GGE biplot was used to graphically display genotype stability and GE interaction under various test environments. The GGE biplot depicted more than 60% of the total variability. Thus, the biplot can safely be interpreted as an effective graphic representation of MET data variability, and the correlations between the two environments are reliable [16]. GGE biplot was performed on six environments (E2, E3, E4, E5, E6, and E7) in this study; E0 and E1 with low heritability were excluded because they accounted for less than 60% variability when included as described in other studies [46].

Marchouch (E2, E3, and E4) and Terbol (E5, E6, and E7) environments were correlated with an angle less than 90° in this study [16]. Terbol-2018/19-no herbicide treatment (E7) was the most discriminating environment, and the least discriminating environments were those treated with imazethapyr and metribuzin (E2, E5, and E6). As a result, the genotypes tested in this study were heavily influenced by the location and herbicide treatment. This is because of the warm and dry weather at Marchouch, where a combination of herbicide treatment and environmental conditions affected the accessions. Therefore, the best test environment for screening lentil accessions for the stability of agronomic performance should be in an environment that is less likely to experience stress periods like Terbol.

A mega-environment is defined as a group of environments that share the best set of genotypes in terms of performance repeatability and consistency [47]. The environments within the same mega-environments (ME1 and ME2) in our study were consistent with the climatic conditions. Fava beans [22] and sorghum [46] yielded comparable results. This confirms that the GE interaction was influenced more by the climatic conditions of the location than by the herbicide treatment.

According to Yan et al. [47], the most responsive genotypes may have the highest or lowest seed yield per plant (SY), but the ideal winning genotype has a high mean yield and high stability [48]. The GGE biplot ranking of genotypes in this study revealed that IG195, IG156635, and IG4605 were the winning accessions with the highest adaptability in ME1 and ME2. Several studies have used the GGE biplot method to identify ideal genotypes in specific environments, including maize [20], barley [49], wheat [50], chickpeas [51], peas [52], and lentils [53]. Our findings were consistent with the ranking of cultivar superiority, which identified the same three winning genotypes and ranked them among the top 15. This supported the findings of Lin and Binns, Makanda et al., and Shiringani and Shimelis [37,38,54] regarding the ability of cultivar superiority to select the genotypes with a combined ability of high stability and yield.

However, IG195, IG4605, and IG156635, the most adapted accessions in ME1 and ME2, were not considered stable using the biplot ranking. Yan and Rajcan [48] reported that an ideal genotype has a high mean yield and high stability across environments. A genotype may be highly stable across the test environments but low yielding or vice versa. In this study, the ranking biplot identified IG70056 (38) as having a high yield as well as being highly stable. Other stability parameters, such as Cultivar superiority and the Shukla parameter, ranked IG70056 (38) among the top ten stable lines included.

## 4. Materials and Methods

### 4.1. Materials and Experiments

**Figure 4:**
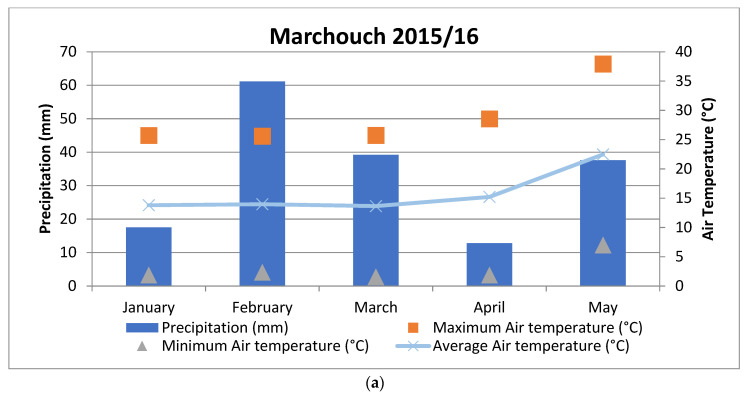

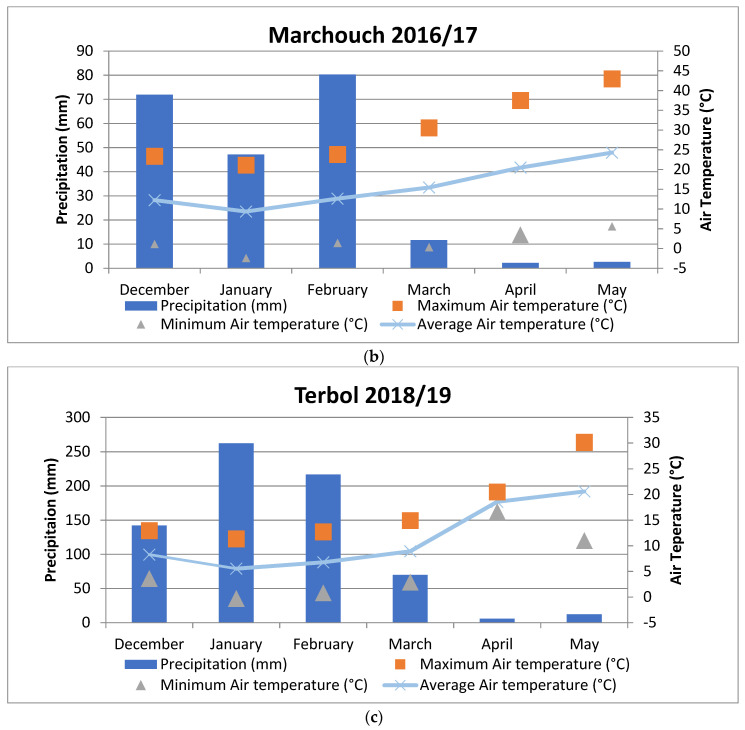
Precipitation (mm), average of minimum and maximum air temperature at Terbol and Marchouch during the three cropping seasons; (**a**) Marchouch 2015/16; (**b**) Marchouch 2016/17, and (**c**) Terbol 2018/19.

In eight separate experiments from 2015 to 2019 under three herbicide treatments, imazethapyr: 75 (g a.i. ha^−1^); metribuzin: 210 (g a.i. ha^−1^) and without herbicide treatment at two locations; Marchouch, Morocco (33.56° N, 6.69° W) and Terbol, Lebanon (33.81° N, 35.98° E); 42 lentils accessions with varying degrees of tolerance to either imazethapyr or metribuzin were selected (unpublished data) and evaluated again (Table 6). Each experiment represents a unique environment resulting from the interaction of seasons, locations, and herbicide treatments. The validation trials and their environments are described in Table 7, and the weather conditions are shown in Figure 4.

The experiments were planted in early December at Terbol and mid-December at Marchouch, and both were harvested in late May. The experiment was led out in an alpha lattice design with two replications with a plot size of 1 row, 1(m) length, 0.3(m) width, and 40 seeds per plot. Herbicides were applied during the pre-flowering stage (5th–6th node stage, 10–15 cm plant height). Except for the post-emergence herbicide treatments, the following agronomic practices were used to raise a successful crop. Trials were conducted in rotation with bread wheat (*Triticum aestivum* L.); soil fertilization with NPK 15-15-15 at 250 kg ha^−1^applied at the pre-sowing stage; pre-emergence application of pendimethalin at 1200 (g a.i. ha^−1^) followed by three manual weedings from the pre-emergence stage until the flowering stage to control seasonal weeds; lambda-cyhalothrin at 40 (g a.i. ha^−1^) and a combination of thiamethoxam and acetamiprid at 200 (g a.i. ha^−1^) were applied to control the sitona leaf weevil (*Sitona crinitus* Herbst) and thrips (*Frankliniella* spp.); a combination of azoxystrobin and difenoconazole at 73 and 46 (g a.i. ha^−1^) were applied to control fungal diseases, especially fusarium wilt (*Fusarium oxysporum* f. sp. *lentils*), and ascochyta blight (*Ascochyta lentils)*.

### 4.2. Recorded Traits

According to Rajendran and Kumar [55], lentil ontology was used to identify the following characteristics:

Days to 50% flowering (DFLR) and 95% maturity data (DMAT) were crop phenology traits measured from the sowing date. Plant height (PH) (cm), number of pods/plant (NPP), number of seeds/plant (NSP), biological yield per plant in g (BY), and seed yield per plant in g (SY) are agronomical and yield traits that were measured on three plants per plot.

### 4.3. Statistical Methods

#### 4.3.1. Variance Analysis

The statistical row-column model was used with Genstat statistical software [56] to assess differences in phenological and agronomic traits among accessions (A) in terms of *p*-values using the Wald statistic. The applied statistical software estimated the best-unbiased values of accessions and combined narrow sense heritability (h^2^) using the residual maximum likelihood (REML). Differences among accessions were assessed using *p*-values using the Wald statistic for each independent environment (E). For DFLR, DMAT, BY, and SY, the narrow sense heritability values (h^2^) were estimated using the residual maximum likelihood method (REML) of Genstat 2019.

#### 4.3.2. Stability Parameters

The following five stability parameters were estimated using Genstat statistical software to compare the performance of genotypes across test environments: (1) Cultivar superiority identifies genotypes with superior performance near the maximum in various environments [54]; (2) the Finlay–Wilkinson parameter identifies lines with general adaptability as those with average stability (bi = 1.0) when associated with high mean yield over tested environment [57], (3) Shukla parameter identifies the stability of the tested genotypes across different environments [58], (4) Static Stability identifies stable genotypes with stable performance under different environments [59] and (5) Wricke’s Ecovalence parameter identifies stability of genotypes based on the GE interaction effects by using the regression approach [60].

#### 4.3.3. GGE Biplot

The GGE scatter biplot was constructed using the best linear unbiased phenotypes (BLUPs) of each accession for each environment to determine the stability of the seed yield per plant across tested environments. To visualize the relationship between the test environments, a vector line was drawn connecting each environment to the biplot origin. The angle between two vectors was used to approximate the correlation between the environments [61,62]. If the angle between the vectors of two environments is less than 90°, the two environments are highly correlated. As a result, the smaller the angle between two vectors, the higher the correlation between the two environments. Furthermore, the biplot depicts mega environments by drawing an ellipse around similar environments in the same sector [15].

The GGE ranking biplot was used to visualize the ranking of accessions based on their SY performance [16]. The ranking biplot abscissa is the line that passes by the biplot origin through the small circle that represents the average of the environments, and its ordinate is the perpendicular line to the abscissa that passes by its origin. The genotype projections to the abscissa represent the average SY estimates. The parallel projections aid in ranking and testing the variability and stability of genotypes based on their predicted mean yield across environments. The farther the projection is away from the axis of the mean environment, the more unstable and variable the genotype under study [15].

## 5. Conclusions

To increase the accuracy of the selection of superior genotypes, the yield and stability of performance across environments should be taken into consideration rather than depending only on the average performance. This study was based on multi-environment trials in which five stability parameters showed inconsistency in ranking the genotypes despite the existence of positive correlations between some of them. Some accessions with higher-than-average yields were classified as unstable, while others with low yields were classified as highly stable. The Static Stability, Finlay–Wilkinson, Wricke’s Ecovalence, and Shukla parameters identified low-yielding genotypes as stable, whereas the GGE biplot and cultivar superiority index ranked the genotypes similarly in terms of yield. The GGE biplot identified IG70056 (38) as a superior line with high and stable yield across years and locations due to its tolerance to imazethapyr and metribuzin. IG4605 (19), IG195 (6), and IG156635 (12) were discovered to be specifically adapted to one mega environment. Furthermore, to avoid the confounding effect, this study recommends conducting herbicide screening trials in environments that do not experience drought periods.

To summarize, in order to develop superior herbicide-tolerant genotypes that are adapted to various mega environments, it is necessary to cross tolerant genotypes that have a stable performance with genotypes adapted to specific environments or that have traits of economic interest.

## Figures and Tables

**Table 1:** Combined analysis for detecting Wald statistics and differences among genotypes, environments, and genotype–environments interaction (GE) and narrow sense heritability (h^2^) for phenological and agronomic traits for the validation trials at Marchouch in 2015/16 and 2016/17 and Terbol in 2018/19.

Trait	Genotypes (G)			Environment (E)			(GE)			h^2^
	d.f.	Wald Statistic	*p*-Value	d.f.	Wald Statistic	*p*-Value	d.f.	Wald Statistic	*p*-Value	
DFLR	41	862.2	<0.001	8	18,556	<0.001	303	904	<0.001	0.93
DMAT	41	583.5	<0.001	8	17,128.9	<0.001	259	418.2	<0.001	0.93
PH	41	125.4	<0.001	5	806.7	<0.001	193	207.6	0.598	0.71
BY	41	337.2	<0.001	8	1995.7	<0.001	298	296.9	0.506	0.21
SY	41	46,522.1	<0.001	8	2160.5	<0.001	293	523.9	<0.001	0.57
NSP	41	125.1	<0.001	2	64.1	<0.001	81	114.5	0.01	-
NPP	41	70	0.003	2	55.7	<0.001	81	95.3	0.15	-

G: Genotypes, df: degree of freedom, h^2^: narrow sense heritability, DFLR: days to 50% flowering, DMAT: days to maturity, PH: plant height, BY: biological yield per plant, SY: seed yield per plant, NPP: number of pods per plant, NSP: number of seeds per plant.

**Table 2:** Wald Statistic and *p*-value performed estimates for detecting differences across genotypes and narrow sense heritability (h^2^) for phenological and agronomic traits.

Environment		DFLR	DMAT	BY	SY
E0	Wald statistic	72.8	33.9	65.8	27.7
	*p*-value	0.035	0.407	0.062	0.62
	h^2^	0.0	0.0	0.0	0.0
E1	Wald statistic	132.1	36.7	92.4	60.4
	*p*-value	0.014	0.303	0.006	0.115
	h^2^	0.4	0.0	0.3	0.0
E2	Wald statistic	112.2	179.1	123.3	203.6
	*p*-value	<0.001	<0.001	0.007	<0.001
	h^2^	0.5	0.6	0.5	0.7
E3	Wald statistic	598.4	262.3	81.7	206.0
	*p*-value	<0.001	<0.001	0.05	<0.001
	h^2^	0.9	0.7	0.3	0.7
E4	Wald statistic	462.2	93.5	87.8	152.4
	*p*-value	<0.001	0.005	0.042	0.002
	h^2^	0.8	0.3	0.3	0.6
E5	Wald statistic	535.9	77.4	66.6	138.4
	*p*-value	<0.001	0.021	0.007	<0.001
	h^2^	0.9	0.3	0.2	0.5
E6	Wald statistic	418.9	54.1	56.6	200.4
	*p*-value	<0.001	0.083	0.053	0.03
	h^2^	0.7	0.1	0.1	0.5
E7	Wald statistic	152.6	66.6	81.3	153.0
	*p*-value	<0.001	0.062	0.081	0.008
	h^2^	0.4	0.2	0.0	0.3

h^2^: narrow sense heritability, DFLR: days to 50% flowering, DMAT: days to maturity, BY: biological yield per plant, SY: seed yield per plant.

**Table 3:** Mean ± Standard error (SE) and ranges for different traits in diverse environments.

Environment		DFLR	DMAT	BY (g/Plant)	SY (g/Plant)
E0	Range	98–118	133–167	0.3–2.4	0.0–0.4
	Mean ± SE	104 ± 0.67	141 ± 2.14	0.9 ± 0.11	0.1 ± 0.01
E1	Range	88–107	141–171	−0.2–6.8	−0.2–1.7
	Mean ± SE	100 ± 0.93	158 ± 4.4	2.4 ± 0.42	0.3 ± 0.21
E2	Range	64–74	96–106	1.6–5.4	0.4–2.1
	Mean ± SE	70 ± 0.17	102 ± 0.61	3.9 ± 0.08	1.1 ± 0.04
E3	Range	52–74	89–106	2.3–6.3	0.6–2.7
	Mean ± SE	63 ± 0.63	98 ± 0.33	4.5 ± 0.12	1.5 ± 0.03
E4	Range	52–73	89–105	2.6–6.4	0.7–2.5
	Mean ± SE	63 ± 0.45	97 ± 1.33	5.1 ± 0.1	1.6 ± 0.04
E5	Range	91–119	134–143	1.1–5.9	−0.0–2.2
	Mean ± SE	104 ± 0.56	139 ± 0.72	3.4 ± 0.37	0.8 ± 0.10
E6	Range	90.3–114	120–140	1.8–7.2	0.4–3.0
	Mean ± SE	97 ± 0.26	129 ± 0.63	3.7 ± 0.25	1.4 ± 0.04
E7	Range	91–107	118–138	1.7–12.6	0.8–4.2
	Mean ± SE	96 ± 1.76	127 ± 1.5	4.8 ± 0.25	2.0 ± 0.13

SE: Standard error, DFLR: days to 50% flowering, DMAT: days to maturity, BY: biological yield per plant, SY: seed yield per plant.

**Table 4:** Five Stability parameters for grain yield of selected lentil accessions and their rankings in eight different environments.

Accession Number	Accession Name	Cultivar Superiority (CS)		Static Stability (SS)		Wricke’s Ecovalence (WE)		Shukla (SH)		Finlay and Wilkinson (FW)	
		CS	R_CS_	SS	R_SS_	WE	R_WE_	SH	R_SH_	FW	R_FW_
1	IG1455	1.63	40	**0.12**	4	1.72	30	0.35	35	**0.82**	2
2	IG2445	1.35	36	**0.12**	2	1.06	26	0.20	27	**1.01**	5
3	IG257	1.68	42	**0.19**	7	**0.12**	2	**0.02**	2	0.67	12
4	IG918	**0.80**	8	**0.12**	3	0.54	11	0.10	14	**1.34**	7
5	IG5626	**0.74**	7	0.51	28	0.66	13	0.13	17	1.26	32
6	IG195	**0.41**	1	1.30	41	2.83	40	0.58	41	1.62	41
7	IG462	1.35	35	0.40	22	1.00	24	0.20	28	0.98	16
8	IG590	1.17	27	0.82	39	1.92	34	0.39	38	1.12	36
9	IG857	1.26	30	0.62	33	1.32	28	0.24	29	0.86	33
10	IG156514	1.14	26	0.26	12	**0.19**	4	**0.03**	4	1.00	19
11	IG156633	0.98	19	0.48	26	**0.47**	8	0.09	12	1.13	30
12	IG156635	**0.48**	3	1.67	42	5.19	42	0.92	42	1.60	42
13	IG156648	**0.74**	6	0.63	34	0.77	16	0.09	11	1.42	37
14	IG156656	0.88	12	0.60	31	0.89	20	0.15	22	1.23	34
15	IG156771	1.21	28	0.48	25	0.95	21	0.19	26	1.03	25
16	IG2684	1.03	22	0.37	19	**0.53**	10	0.09	13	1.05	23
17	IG4400	0.99	20	**0.15**	5	2.15	37	0.14	19	**1.25**	3
18	IG4401	1.25	29	0.47	24	0.72	14	0.15	21	0.86	29
19	IG4605	0.90	14	0.83	40	2.86	41	0.47	39	1.55	27
20	IG5244	0.95	18	0.46	23	**0.36**	6	**0.07**	7	1.11	31
21	IG5562	**0.45**	2	0.79	37	1.42	29	0.29	32	1.56	38
22	IG5588	1.08	25	0.31	13	0.78	17	0.14	20	1.06	13
23	IG69492	1.06	24	0.36	17	**0.09**	1	**0.01**	1	1.06	28
24	IG70079	**0.82**	10	0.77	36	1.79	31	0.28	31	1.42	35
25	IG71366	1.05	23	0.36	16	2.08	36	0.29	33	**1.16**	6
26	IG75929	1.32	33	**0.22**	9	**0.15**	3	**0.03**	3	0.88	15
27	IG75932	0.85	11	0.57	30	1.97	35	0.36	36	1.25	21
28	IG76251	**0.80**	9	0.38	21	**0.48**	9	**0.07**	8	1.30	24
29	IG114663	1.44	37	**0.22**	8	**0.25**	5	**0.05**	5	0.84	11
30	IG114670	0.93	16	**0.18**	6	2.17	38	**0.07**	9	**1.25**	1
31	IG114703	0.91	15	0.81	38	1.27	27	0.26	30	1.10	39
32	IG115370	1.27	31	0.35	15	**0.39**	7	**0.08**	10	0.96	22
33	IG117684	1.31	32	0.38	20	1.92	33	0.31	34	**1.07**	8
34	ILL8009	1.35	34	0.56	29	1.88	32	0.37	37	1.08	18
35	IG138106	1.55	39	0.35	14	0.58	12	0.12	15	0.79	20
36	ILX87075	1.66	41	**0.10**	1	0.99	23	0.14	18	**0.76**	4
37	L24	0.90	13	0.61	32	2.50	39	0.51	40	1.25	14
38	IG70056	**0.58**	4	0.73	35	0.74	15	**0.05**	6	1.44	40
39	2009S 96568-1	**0.58**	5	0.37	18	0.80	18	0.16	23	1.64	17
40	IG156801	1.54	38	**0.23**	10	1.04	25	0.16	24	**0.81**	9
41	010S 96130-1	0.93	17	0.51	27	0.99	22	0.18	25	1.29	26
42	010S 96155-2	1.03	21	0.26	11	0.84	19	0.13	16	**1.14**	10

Highlighted in bold are the 10 most stable accessions, R_CS_: Ranking of accessions based on Cultivar Superiority, R_SS_: Ranking of accessions based on Static Stability, R_WE_: Ranking of accessions based on Wricke’s Ecovalence, R_FW_: Ranking of accessions based on Finlay–Wilkinson and R_SH_: Ranking of accessions based on Shukla.

**Table 5:** Spearman’s coefficients of rank correlation for five stability parameters analyzed for grain yield of lentil accessions tested in different environments.

	Cultivar Superiority	Finlay and Wilkinson	Shukla	Static Stability
Finlay and Wilkinson	−0.57 ***	-		
Shukla	−0.17	0.15	-	
Static Stability	−0.60 ***	0.87 ***	0.52 ***	-
Wricke’s Ecovalence	−0.24	−0.01	0.87 ***	0.39 *

*** *p* < 0.001, * *p* < 0.05.

**Table 6:** List of selected lentil accessions and their tolerance to imazethapyr and metribuzin in validation trials based on a preliminary trial led at Marchouch-2014/15 (unpublished data).

No	Accessions	Tolerance to Imazethapyr (75 g a.i. ha^−1^)	Tolerance to Metribuzin (210 g a.i. ha^−1^)
1	IG1455	Moderately Tolerant	Moderately Tolerant
2	IG2445	Moderately Tolerant	Tolerant
3	IG257	Moderately Tolerant	Highly Tolerant
4	IG918	Moderately Tolerant	Tolerant
5	IG5626	Moderately Tolerant	Tolerant
6	IG195	Moderately Tolerant	Highly Tolerant
7	IG462	Moderately Tolerant	Highly Tolerant
8	IG590	Moderately Tolerant	Tolerant
9	IG857	Moderately Tolerant	Tolerant
10	IG156514	Moderately Tolerant	Tolerant
11	IG156633	Moderately Tolerant	Moderately Tolerant
12	IG156635	Moderately Tolerant	Tolerant
13	IG156648	Moderately Tolerant	Tolerant
14	IG156656	Moderately Tolerant	Tolerant
15	IG156771	Moderately Tolerant	Tolerant
16	IG2684	Moderately Tolerant	Moderately Tolerant
17	IG4400	Moderately Tolerant	Tolerant
18	IG4401	Moderately Tolerant	Tolerant
19	IG4605	Moderately Susceptible	Tolerant
20	IG5244	Moderately Tolerant	Tolerant
21	IG5562	Moderately Tolerant	Moderately Tolerant
22	IG5588	Moderately Tolerant	Tolerant
23	IG69492	Moderately Tolerant	Tolerant
24	IG70079	Moderately Tolerant	Tolerant
25	IG71366	Moderately Tolerant	Highly Tolerant
26	IG75929	Moderately Tolerant	Tolerant
27	IG75932	Moderately Tolerant	Tolerant
28	IG76251	Moderately Tolerant	Tolerant
29	IG114663	Moderately Tolerant	Tolerant
30	IG114670	Moderately Tolerant	Tolerant
31	IG114703	Moderately Tolerant	Tolerant
32	IG115370	Moderately Susceptible	Tolerant
33	IG117684	Moderately Tolerant	Tolerant
34	ILL8009	Tolerant	Tolerant
35	IG138106	Moderately Tolerant	Tolerant
36	ILX87075	Moderately Tolerant	Tolerant
37	L24	Moderately Tolerant	Tolerant
38	IG70056	Moderately Tolerant	Tolerant
39	2009S 96568-1	Moderately Tolerant	Tolerant
40	IG156801	Moderately Tolerant	Highly Tolerant
41	010S 96130-1	Moderately Tolerant	Tolerant
42	010S 96155-2	Moderately Tolerant	Tolerant

**Table 7:** Specifications and details of the various environments tested for lentil screening.

Environment	Environment (Location-Cropping Season-Treatment)	Soil Type	Rainfall (mm)	Air Temperature (°C)		
				AVG	AVG Min	AVG Max
E0	Marchouch-2015/16-metribuzin at 210 g a.i. ha^−1^	Vertisols and silty clay	168	18.24	6.71	34.03
E1	Marchouch-2015/16-no herbicide treatment					
E2	Marchouch-2016/17-imazethapyr at 75 g a.i. ha^−1^	Vertisols and silty clay	211	14.05	−2.4	42.99
E3	Marchouch-2016/17-metribuzin at 210 g a.i. ha^−1^					
E4	Marchouch-2016/17-No Herbicide Treatment					
E5	Terbol-2018/19-imazethapyr at 75 g a.i. ha^−1^	Clay loam	810	11.7	−0.28	32.3
E6	Terbol-2018/19-metribuzin at 210 g a.i. ha^−1^					
E7	Terbol-2018/19-no herbicide treatment					

## Data Availability

Data is contained within the article.

## References

[1] Cokkizgin A., Shtaya M.J.Y. (2013). Lentil: Origin, Cultivation Techniques, Utilization and Advances in Transformation. Agric. Sci..

[2] Johnson N., Johnson C.R., Thavarajah P., Kumar S., Thavarajah D. (2020). The roles and potential of lentil prebiotic carbohydrates in human and plant health. Plants People Planet.

[3] Erskine W., Sarker A., Kumar S. (2011). Crops that feed the world 3. Investing in Lentil Improvement toward a Food Secure World. Food Secur..

[4] FAOSTAT Statistical Database. www.faostat.fao.org.

[5] Rubiales D., Fernández-Aparicio M. (2012). Innovations in parasitic weeds management in legume crops. A Review. Agron. Sustain. Dev..

[6] Sharma S.R., Singh S., Aggarwal N., Kaur J., Gill R.K., Kushwah A., Patil S.B., Kumar S. (2018). Genetic Variation for Tolerance to Post-Emergence Herbicide, Imazethapyr in Lentil (*Lens culinaris* Medik.). Arch. Agron. Soil Sci..

[7] Balech R., Maalouf F., Patil S.B., Hejjaoui K., Abou Khater L., Rajendran K., Rubiales D., Kumar S. (2022). Evaluation of Performance and Stability of New Sources for Tolerance to Post-Emergence Herbicides in Lentil (*Lens culinaris* ssp. *culinaris* Medik.). Crop Pasture Sci..

[8] Gaur P., Jukanti A., Samineni S., Chaturvedi S., Singh S., Tripathi S., Singh I., Singh G., Das T., Aski M. (2013). Large Genetic Variability in Chickpea for Tolerance to Herbicides Imazethapyr and Metribuzin. Agronomy.

[9] Redlick C., Syrovy L.D., Duddu H.S.N., Benaragama D., Johnson E.N., Willenborg C.J., Shirtliffe S.J. (2017). Developing an Integrated Weed Management System for Herbicide-Resistant Weeds Using Lentil (*Lens culinaris*) as a Model Crop. Weed Sci..

[10] Yenish J.P., Yadav S.S., Redden B., Chen W., Sharma B. (2007). Weed Management in Chickpea. Chickpea Breeding and Management.

[11] Sharma S.R., Singh S., Aggarwal N., Kushwah A., Kumar S. (2017). Inherent Variability among Different Lentil (*Lens culinaris* Medik.) Genotypes against Tolerance to Metribuzin Herbicide. Biochem. Cell. Arch..

[12] Oliveira M.C., Feist D., Eskelsen S., Scott J.E., Knezevic S.Z. (2017). Weed Control in Soybean with Preemergence- and Postemergence-Applied Herbicides. Crop Forage Turfgrass Manag..

[13] Johnson G.A., Hoverstad T.R., Greenwald R.E. (1998). Integrated Weed Management Using Narrow Corn Row Spacing, Herbicides, and Cultivation. Agron. J..

[14] Slinkard A.E., Vanderberg A., Holm F.A. (2007). Lentil Plants Having Increased Resistance to Imidazolinone Herbicides. U.S. Patent.

[15] Turk Z., Kendal E. (2017). Practice of AMMI and GGE Biplot Analysis of Lentil Genotypes Assessment in Multi-Environment Trials. Philipp. J. Crop Sci..

[16] Yan W., Hunt L.A., Sheng Q., Szlavnics Z. (2000). Cultivar Evaluation and Mega-environment Investigation Based on the GGE Biplot. Crop Sci..

[17] Sayar M.S., Anlarsal A.E., Basbag M. (2013). Genotype-Environment Interactions and Stability Analysis for Dry-Matter Yield and Seed Yield in Hungarian Vetch (*Vicia pannonica* Crantz.). Turk. J. Field Crops.

[18] De Leon N., Jannink J.L., Edwards J.W., Kaeppler S.M. (2016). Introduction to a Special Issue on Genotype by Environment Interaction. Crop Sci..

[19] Sabaghnia N., Dehghani H., Sabaghpour S.H. (2008). Graphic Analysis of Genotype by Environment Interaction for Lentil Yield in Iran. Agron. J..

[20] Fan X.M., Kang M.S., Chen H., Zhang Y., Tan J., Xu C. (2007). Yield Stability of Maize Hybrids Evaluated in Multi-Environment Trials in Yunnan, China. Agron. J..

[21] Sayar M.S., Han Y. (2015). Determination of Seed Yield and Yield Components of Grasspea (*Lathyrus sativus* L.) Lines and Evaluations Using GGE Biplot Analysis Method. J. Agric. Sci..

[22] Abou-Khater L., Maalouf F., Jighly A., Rubiales D., Kumar S. (2022). Adaptability and Stability of Faba Bean (*Vicia faba* L.) Accessions under Diverse Environments and Herbicide Treatments. Plants.

[23] Dehghani H., Sabaghpour S.H., Sabaghnia N. (2008). Genotype × Environment Interaction for Grain Yield of Some Lentil Genotypes and Relationship among Univariate Stability Statistics. Span. J. Agric. Res..

[24] Gauch J., Hugh G. (2006). Statistical Analysis of Yield Trials by AMMI and GGE. Crop Sci..

[25] Karimizadeh R., Mohammadi M., Sabaghni N., Mahmoodi A.A., Roustami B., Seyyedi F., Akbari F. (2013). GGE Biplot Analysis of Yield Stability in Multi-Environment Trials of Lentil Genotypes under Rainfed Condition. Not. Sci. Biol..

[26] Abou-Khater L., Maalouf F., Patil S.B., Balech R., Rubiales D., Kumar S. (2021). Identification of Tolerance to Metribuzin and Imazethapyr Herbicides in Faba Bean (*Vicia faba* L.). Crop Sci..

[27] Royuela M., Gonzalez A., Gonzalez E.M., Arrese-Igor C., Aparicio-Tejo P.M., Gonzalez-Murua C. (2000). Physiological Consequences of Continuous, Sublethal Imazethapyr Supply to Pea Plants. J. Plant Physiol..

[28] Choukri H., Hejjaoui K., El-Baouchi A., Haddad N.E., Smouni A., Maalouf F., Thavarajah D., Kumar S. (2020). Heat and Drought Stress Impact on Phenology, Grain Yield, and Nutritional Quality of Lentil (*Lens culinaris* Medikus). Front. Nutr..

[29] Rani A., Devi P., Jha U.C., Sharma K.D., Siddique K.H., Nayyar H. (2020). Developing Climate-Resilient Chickpea Involving Physiological and Molecular Approaches with a Focus on Temperature and Drought Stresses. Front. Plant Sci..

[30] Maalouf F., Nachit M., Ghanem M.E., Singh M. (2015). Evaluation of Faba Bean Breeding Lines for Spectral Indices, Yield Traits and Yield Stability under Diverse Environments. Crop Pasture Sci..

[31] Taran B., Warkentin T.D., Vandenberg A., Holm F.A. (2010). Variation in Chickpea Germplasm for Tolerance to Imazethapyr and Imazamox Herbicides. Can. J. Plant Sci..

[32] Mohammed A., Tesso B., Ojiewo C., Ahmed S. (2019). Assessment of Genetic Variability and Heritability of Agronomic Traits of Ethiopian Chickpea (*Cicer arietinum* L.) Landraces. Black Sea J. Agric..

[33] Bicer B.T., Sakar D. (2006). Evaluation of Lentil (*Lens culinaris* Medik.) Local Varieties in Southeastern Anatolia, Turkey. Bulg. J. Agric. Sci..

[34] Mohebodini M., Dehghani H., Hossain S.S. (2006). Stability of Performance in Lentil (*Lens culinaris* Medik) Genotypes in Iran. Euphytica.

[35] Yadav S.S., Verma A.K., Rizvi A.H., Singh D., Kumar J., Andrews M. (2010). Impact of Genotype× Environment Interactions on the Relative Performance of Diverse Groups of Chickpea (*Cicer arietinum* L.) Cultivars. Arch. Agron. Soil Sci..

[36] Adugna A. (2007). Assessment of Yield Stability in Sorghum. Afr. Crop Sci. J..

[37] Shiringani R.P., Shimelis H.A. (2011). Yield Response and Stability among Cowpea Genotypes at Three Planting Dates and Test Environments. Afr. J. Agric. Res..

[38] Makanda I., Tongoona P., Derera J., Sibiya J., Fato P. (2010). Combining Ability and Cultivar Superiority of Sorghum Germplasm for Grain Yield across Tropical Low-and Mid-Altitude Environments. F Crop Res..

[39] Fasahat P. (2015). An Overview on the Use of Stability Parameters in Plant Breeding. Biom. Biostat. Int. J..

[40] Ramazani S.H.R., Tajalli H., Ghoudsi M. (2016). Evaluation of Grain Yield Stability of Superior Triticale Genotypes. Bulg. J. Agric. Sci..

[41] Mustapha M., Bakari H.R. (2014). Statistical Evaluation of Genotype by Environment Interactions for Grain Yield in Millet (*Penniisetum glaucum* (L.) R. Br.). Int. J. Eng. Sci..

[42] Westcott B. (1986). Some Methods of Analysing Genotype—Environment Interaction. Heredity.

[43] Roy S., Islam M.A., Sarker A., Malek M.A., Rafii M.Y., Ismail M.R. (2013). Determination of Genetic Diversity in Lentil Germplasm Based on Quantitative Traits. Aust. J. Crop Sci..

[44] Stewart C.L., Nurse R.E., Hamill A.S., Sikkema P.H. (2010). Environment and Soil Conditions Influence Pre-and Postemergence Herbicide Efficacy in Soybean. Weed Technol..

[45] Stewart C.L., Soltani N., Nurse R.E., Hamill A.S., Sikkema P.H. (2012). Precipitation Influences Pre-and Post-Emergence Herbicide Efficacy in Corn. Am. J. Plant Sci..

[46] Rakshit S., Ganapathy K.N., Gomashe S.S., Rathore A., Ghorade R.B., Kumar M.N., Patil J.V. (2012). GGE Biplot Analysis to Evaluate Genotype, Environment and Their Interactions in Sorghum Multi-Location Data. Euphytica.

[47] Yan W., Kang M.S., Ma B., Woods S., Cornelius P.L. (2007). GGE Biplot vs. AMMI Analysis of Genotype-by-Environment Data. Crop Sci..

[48] Yan W., Rajcan I. (2002). Biplot Analysis of Test Sites and Trait Relations of Soybean in Ontario. Crop Sci..

[49] Dehghani H., Ebadi A., Yousefi A. (2006). Biplot Analysis of Genotype by Environment Interaction for Barley Yield in Iran. Agron. J..

[50] Kaya Y., Akçura M., Taner S. (2006). GGE-Biplot Analysis of Multi-Environment Yield Trials in Bread Wheat. Turk. J. Agric. For..

[51] Erdemci I. (2018). Investigation of Genotype × Environment Interaction in Chickpea Genotypes Using AMMI and GGE Biplot Analysis. Turk. J. Field Crops.

[52] Rubiales D., Osuna-Caballero S., González-Bernal M.J., Cobos M.J., Flores F. (2021). Pea Breeding Lines Adapted to Autumn Sowings in Broomrape Prone Mediterranean Environments. Agronomy.

[53] Rubiales D., Moral A., Flores F. (2021). Heat Waves and Broomrape Are the Major Constraints for Lentil Cultivation in Southern Spain. Agronomy.

[54] Lin C.-S., Binns M.R. (1988). A Superiority Measure of Cultivar Performance for Cultivar × Location Data. Can. J. Plant Sci..

[55] Kumar S., Rajendran K. Lentil Ontology. https://cropontology.org/term/CO_339:ROOT.

[56] Goedhart P.W., Thissen J.T.N.M. (2018). Biometris Genstat Procedure Library Manual.

[57] Finlay K.W., Wilkinson G.N. (1963). The Analysis of Adaptation in a Plant-Breeding Programme. Aust. J. Agric. Res..

[58] Shukla G.K. (1972). Some Statistical Aspects of Partitioning Genotype-Environmental Components of Variability. Heredity.

[59] Becker H.C., Leon J. (1988). Stability Analysis in Plant Breeding. Plant Breed..

[60] Wricke G. (1962). Uber Eine Methode zur Erfassung der Okologischen Streubreite in Feldverzuchen. Z. Pflanzenzuchtg.

[61] Yan W., Tinker N.A. (2006). Biplot Analysis of Multi-Environment Trial Data: Principles and Applications. Can. J. Plant Sci..

[62] Kaya Y., Turkoz M. (2016). Evaluation of Genotype by Environment Interaction for Grain Yield in Durum Wheat Using Non-Parametric Stability Statistics. Turk. J. Field Crops.

